# Exploring the expression and prognostic roles of LAD1 in lung adenocarcinoma

**DOI:** 10.1038/s41598-025-33277-z

**Published:** 2025-12-21

**Authors:** Sufen Wang, Banghong Qiang, Xiaoyan Xu, Yan Fang, Yinhua Liu, Wei Zhang, Xi Huang

**Affiliations:** 1 Department of Pathology, The First Affiliated Hospital of Wannan Medical College (Yijishan Hospital of Wannan Medical College), Wuhu, Anhui China; 2 Department of Ultrasound Medicine, Wuhu Hospital, East China Normal University (The Second People’s Hospital, Wuhu), Wuhu, Anhui China; 3 School of Medical Imageology, Wannan Medical College, Wuhu, Anhui China

**Keywords:** Lung adenocarcinoma (LUAD), Single-cell RNA sequencing (scRNA-seq), LAD1, Migration, Invasion, Lung cancer, Cancer, Drug discovery, Genetics, Molecular biology, Biomarkers

## Abstract

Lung adenocarcinoma (LUAD) is a common subtype of non-small cell lung cancer (NSCLC) with a poor prognosis. To identify novel biomarkers and understand the underlying mechanisms in LUAD, we conducted a comprehensive analysis using single-cell and bulk RNA sequencing data. Our study focused on LAD1, a basement membrane filament protein that has been implicated in tumorigenesis in various cancers. We analyzed scRNA-seq data from 10 LUAD patients and identified nine cell subgroups, with LAD1 specifically expressed in cancer cells. Further analysis revealed significant correlations between LAD1 and genes associated with LUAD progression, including SFTPB, S100A6, CEACAM6, KRT19, S100A10, ANXA2, S100A11, and CAPN2. We investigated the effects of altered LAD1 expression on differentially expressed genes (DEGs), biological processes, and signaling pathways. Furthermore, we collected cancer tissue and corresponding adjacent tissue samples from 36 LUAD patients, used immunohistochemical staining to detect LAD1 expression, and analyzed its correlation with clinicopathological characteristics. We knocked down the expression of LAD1 in A549 cells using siRNA, and detected changes in LUAD cell migration and invasion ability through scratch healing assay and Transwell assay. Our results indicated diverse effects of LAD1 at both the single-cell and whole tumor levels, with a convergence on processes associated with tumor progression. In lung adenocarcinoma tissues, LAD1 was significantly upregulated, particularly in the cancer cell subgroup within tumors. Immunohistochemical results showed that LAD1 was highly expressed in LUAD, and knocking down LAD1 could inhibit LUAD cell migration and invasion. Pan-cancer analyses demonstrated LAD1 as an independent prognostic factor for overall survival in LUAD. Moreover, we developed a nomogram model incorporating LAD1 expression and clinical parameters, which demonstrated good predictive performance. The high expression of LAD1 in cancer cells, its associations with LUAD-related genes, and its links to biological processes and pathways suggest its potential biological relevance and that it merits further investigation. Overall, this study provides insights into LUAD and supports LAD1 as a gene worthy of further investigation.

**Supplementary Information:**

The online version contains supplementary material available at 10.1038/s41598-025-33277-z.

## Introduction

Lung cancer is a leading cause of morbidity and mortality worldwide^1^, with non-small cell lung cancer (NSCLC) and small cell lung cancer (SCLC) being the two main types. NSCLC, particularly lung adenocarcinoma (LUAD), accounts for the majority of lung cancer cases and poses a significant global health threat^2^. LUAD is often diagnosed at advanced stages, resulting in poor prognosis^3^. Treatment options for LUAD include surgery, chemotherapy, radiotherapy, and immunotherapy, with targeted therapies showing promising results by inhibiting specific tyrosine kinase pathways such as EGFR, ALK, and ROS1^4^. However, the genetic profiles of tumors can vary substantially between individuals, necessitating personalized therapeutic approaches based on gene expression patterns. Several genes have been identified as associated with LUAD tumorigenesis and prognosis, including those involved in signaling transduction, tumor microenvironment, and metabolism^5–7^. Nonetheless, the discovery of additional prognostic genes holds the potential to further enhance therapeutic outcomes in LUAD.

LAD1 (Ladinin 1) is a protein-coding gene responsible for encoding a basement membrane filament^8^. Its role involves stabilizing the association between epithelial and mesenchyme layers^9^. LAD1 has generally been implicated as a favorable factor in tumorigenesis. In colorectal cancer, upregulated LAD1 has been identified as a significant hazard prognostic factor associated with enhanced metastasis^10^. Similar associations have been observed in prostate cancer and breast cancer, where LAD1 expression correlates with poor prognosis and drug resistance^11–13^. In LUAD, previous studies have suggested that LAD1 promotes cell cycle progression, migration^14,15^, and contributes to an immunosuppressive microenvironment, potentially hampering immunotherapy effectiveness^16^. Despite these reports, a comprehensive understanding of LAD1’s expression patterns, correlations with other genes, and potential regulatory networks in different cell types within LUAD tumors remains limited. Addressing these gaps requires the application of single-cell RNA sequencing (scRNA-seq), which enables the exploration of gene expression patterns at the single-cell level and the characterization of distinct cell subpopulations within tumors.

In this study, we employed scRNA-seq to investigate the expression distribution of LAD1 across various cell subgroups in LUAD. Our analysis revealed specific expression of LAD1 in cancer cells compared to other cell types.We assessed the impact of altered LAD1 expression on differentially expressed genes (DEGs), biological processes, and signaling pathways at both the single-cell and whole-tumor levels. Notably, LAD1 exhibited diverse effects, highlighting the importance of examining gene functions in both the context of entire tumors and specific cell subsets. The immunohistochemical findings revealed that LAD1 exhibited high expression levels in LUAD, and that silencing LAD1 could suppress LUAD cell migration and invasion.Furthermore, we examined the prognostic significance of LAD1 in LUAD and identified it as an independent hazard factor for overall survival. Additionally, we developed a nomogram model incorporating LAD1 expression and other clinical parameters, which was validated using actual prognosis data.

## Materials and methods

### General information of datasets

We obtained the single-cell RNA sequencing (scRNA-seq) data of lung adenocarcinoma (LUAD) from the Gene Expression Omnibus (GEO) database (GSE136246) [https://www.ncbi.nlm.nih.gov/geo/]. The mRNA and protein profile data of normal tissues were downloaded from the Genotype Tissue Expression (GTEx) database [https://gtexportal.org/] and Human Protein Atlas (HPA) databases [https://www.proteinatlas.org/], respectively. The mRNA profile data of various cancer cell lines were obtained from the Cancer Cell Line Encyclopedia (CCLE) database [https://portals.broadinstitute.org/ccle/]. For the analysis of LUAD and other cancers, we retrieved the expression and clinical information from The Cancer Genome Atlas (TCGA) database [https://portal.gdc.cancer.gov/] (Table 1). Additionally, to validate our findings, we downloaded three independent cohorts (GSE72094, GSE41271, and GSE30219) from the GEO database. To facilitate downstream analysis, patients in the TCGA-LUAD cohort were stratified into LAD1_high and LAD1_low groups based on the median expression value of LAD1. This dichotomization allowed for comparative analyses of clinical characteristics and survival outcomes.

**Table 1:** Clinical characteristics of the TCGA-LUAD cohort.

Characteristics	LAD1_high (*N* = 287)	LAD1_low (*N* = 228)	*P*-value
gender			
MALE	141 (49.1%)	97 (42.5%)	0.162
FEMALE	146 (50.9%)	131 (57.5%)	
Stage			
NA	4 (1.4%)	4 (1.8%)	0.117
I	145 (50.5%)	130 (57.0%)	
II	71 (24.7%)	51 (22.4%)	
III	56 (19.5%)	28 (12.3%)	
IV	11 (3.8%)	15 (6.6%)	
KRAS_mut			
NA	152 (53.0%)	133 (58.3%)	0.159
N	90 (31.4%)	72 (31.6%)	
Y	45 (15.7%)	23 (10.1%)	
AGE			
NA	16 (5.6%)	15 (6.6%)	0.443
<=65	124 (43.2%)	109 (47.8%)	
> 65	147 (51.2%)	104 (45.6%)	
LAD1			
Mean (SD)	232 (118)	80.0 (32.7)	< 0.001
Median [Min, Max]	195 [129, 943]	85.5 [0.433, 129]	

TCGA, The Cancer Genome Atlas; LUAD, Lung Adenocarcinoma; NA, not applicable. LAD1_high and LAD1_low groups were defined based on the median expression level of LAD1 in the TCGA-LUAD cohort.

### scRNA-seq data analysis

The scRNA-seq data consisted of samples from 10 LUAD patients, which were merged using the “CCA” package in R. We performed doublet removal using the “DoubletFinder” package in R. Cells with fewer than 200 expressed genes and genes expressed in less than 3 cells were filtered out. After quality control, the scRNA-seq data contained expression and metadata from 58,685 cells. Subsequent analyses were performed using the “Seurat” package in R. We identified the top 2000 genes with altered expression and performed Principal Component Analysis (PCA) using these genes. The t-distributed stochastic neighbor embedding (t-SNE) analysis was then conducted using the top 30 Principal Components. Clustering was performed using the “FindCluster” function with a resolution of 0.5, resulting in the identification of 22 clusters. These clusters were annotated using canonical cell type markers, resulting in the identification of 9 cell subgroups. The markers used for annotation included VWF and PECAM1 for endothelial cells, CAPS for epithelial cells, COL1A1, COL1A2, and DCN for fibroblasts, CD2 and CD3D for T cells, CD79a for B cells, CD14 and Lys for myeloid cells, S100A8 and S100A9 for neutrophils, GATA2, TPSAB1, and TPSB2 for mast cells, and EPCAM for cancer cells. The marker genes of these single-cell subpopulations were obtained from the CellMarker 2.0 database [http://117.50.127.228/CellMarker/CellMarkerBrowse.jsp*].* The expression of LAD1 in the scRNA-seq data was visualized using the “FeaturePlot” and “VlnPlot” functions. We divided the cells in the cancer cell subgroup into high and low LAD1 groups based on the median expression of LAD1. The differentially expressed genes (DEGs) between these groups were determined using the “FindMarkers” function with a cutoff of *P* < 0.05. The correlation between LAD1 and the DEGs was analyzed using the “cor” function and visualized with the “corrplot” function in R.

### RNA-sequencing (RNA-seq) data analysis

The TCGA-LUAD cohort was divided equally into high and low LAD1 groups based on the median expression of LAD1. The DEGs between these groups were determined using the “DESeq2” package in R, with a cutoff of *P* < 0.05 and |log2(FoldChange)| > 1. A heatmap showing the relative expressions of the top 50 altered DEGs was generated using the “pheatmap” package in R.

### Gene ontology (GO) and pathway analyses

GO analysis and gene set enrichment analysis (GSEA) were performed using the “clusterProfiler” package in R. Kyoto Encyclopedia of Genes and Genomes (KEGG) analysis was conducted using the Database for Annotation, Visualization, and Integrated Discovery (DAVID). A significance cutoff of *P* < 0.05 was applied, and the results were visualized using the “clusterProfiler” and “ggplot2” packages in R. Pathway analysis was performed using data from the Kyoto Encyclopedia of Genes and Genomes [*KEGG; Kanehisa Laboratories*, www.kegg.jp/kegg/kegg1.html*].*

### Pan-cancer analyses of gene expression and prognosis

The expression of LAD1 in normal tissues was determined using data downloaded from the GTEx and HPA databases. The expression of LAD1 in cancer cell lines was analyzed using data from the CCLE database. The prognostic value of LAD1 in pan-cancer was analyzed using gene expression and clinical information from the TCGA database. Patients in each cancer type were divided into high and low LAD1 groups based on the median expression of LAD1 in the corresponding cancer type. Overall survival (OS), disease-specific survival (DSS), progression-free survival (PFS), and disease-free survival (DFS) were compared between the two groups in each cancer type. Kaplan-Meier survival curves were plotted using the “survival” and “survminer” packages in R. Other results were visualized using the “ggplot2” package in R, with a significance cutoff of *P* < 0.05.

### Immune infiltration analysis

For LUAD patients in TCGA, we divided them into high and low LAD1 groups based on the median expression of LAD1. We analyzed anti-cancer immune responses using data obtained from the Tracking Tumor Immunophenotype (TIP) database. Immune cell infiltration in LUAD was determined using the CIBERSORT analysis. A significance threshold of *P* < 0.05 was applied.

### Cox regression analysis

Univariate Cox analyses were performed to identify significant prognostic factors for LUAD overall survival (OS). Multivariate Cox analysis was then conducted to determine whether these prognostic factors were independent. Both univariate and multivariate Cox analyses were performed using the “coxph” function in the “survival” package in R. A significance threshold of *P* < 0.05 was applied.

### Nomogram analysis

The expression and clinical data of LUAD from TCGA were subjected to nomogram analysis using the “RMS” package in R. The clinical factors included in the nomogram analysis were LAD1 level, gender, TNM stages, KRAS mutations, and age. The prognostic prediction model was validated using 1-, 2-, and 3-year actual OS data.

### Immunohistochemical staining

We randomly selected 36 samples from LUAD patients in our hospital in 2024 for immunohistochemical staining, and collected their clinical and pathological data. LUAD was staged (pTNM) based on postoperative pathology in accordance with the AJCC 8th edition TNM classification. These tissues, after being embedded in paraffin, were sectioned for the staining process. We performed immunohistochemical staining using the Envision two-step method, employing LAD1 polyclonal antibody (NBP1-85945, Novus Biologicals) as the primary antibody. Images of the stained tissues were captured using a Nikon Ci-L microscope. For each slide, five visual fields were randomly chosen for analysis. The semi- quantitative scores were calculated by adding the staining intensity (0 represents no staining, 1 represents weak staining, 2 represents moderate staining, 3 represents strong staining) to the proportion of stained cells (0 represents < 10%, 1 represents 10%-24%, 2 represents 25%-49%, 3 represents ≥ 50%). Scores of 0–1 were considered negative, 2 weakly positive, 3–4 moderately positive, 5–6 strongly positive. A total score greater than 2 was defined as positive. The study was approved by the Ethics Committee of the First Affiliated Hospital of Wannan Medical College (approval number: 2024-RE-195). The requirement for written informed consent was waived by the Ethics Committee of the First Affiliated Hospital of Wannan Medical College due to the retrospective nature of the study. All methods were carried out in accordance with relevant guidelines and regulations, and the study was conducted in accordance with the Declaration of Helsinki.

### Validation of LAD1 knockdown cell model

A LUAD cell line was established with LAD1 silenced using siRNA, and its silencing efficiency was assessed. The LUAD cell line A549 was transfected with siRNA, and after 48 h of transfection, the knockdown efficiency was evaluated via Western blot.

### Scratch test and transwell test

The wound healing assay was performed following the manufacturer’s protocol for the Culture-Insert 2 Well system (Ibidi, 80209). Briefly, single-cell suspensions were obtained through enzymatic digestion and adjusted to a concentration of 3 × 10^4^cells/mL. A total of 70 µL of the suspension was added to each well, ensuring that shaking was avoided to prevent uneven cell distribution. The plates were incubated under standard culture conditions (37 °C, 5% CO₂) for 24 h to allow cell adherence. Subsequently, the Culture-Insert 2 Well was carefully removed using sterile tweezers, and the wells were gently washed before replacing the medium with serum-free DMEM. Cultures were maintained at 37 °C in a 5% CO₂ atmosphere, and wound closure was monitored microscopically at 0, 24, and 48 h.

The invasion assay was conducted according to the manufacturer’s instructions for the Plurigel Matrix, LDEV-Free (Vazyme Biotech Co., Ltd., GL101). Briefly, a cell suspension (3 × 10^4^cells/mL) prepared in serum-free DMEM was seeded into the upper chambers of Transwell inserts (Corning, 3422). The lower chambers were filled with DMEM supplemented with 10% fetal bovine serum (FBS) to serve as a chemoattractant. After incubation for 48 h at 37 °C in 5% CO₂, the non-invading cells were removed, and the membranes were fixed with 4% methanol-free formaldehyde (CST, 47746) for 30 min. The invaded cells were then stained with 0.1% crystal violet for 15 min and imaged under a light microscope.

## Results

### scRNA-seq identified LAD1 as a cancer cell-specific marker in lung adenocarcinoma (LUAD)

**Fig. 1:**
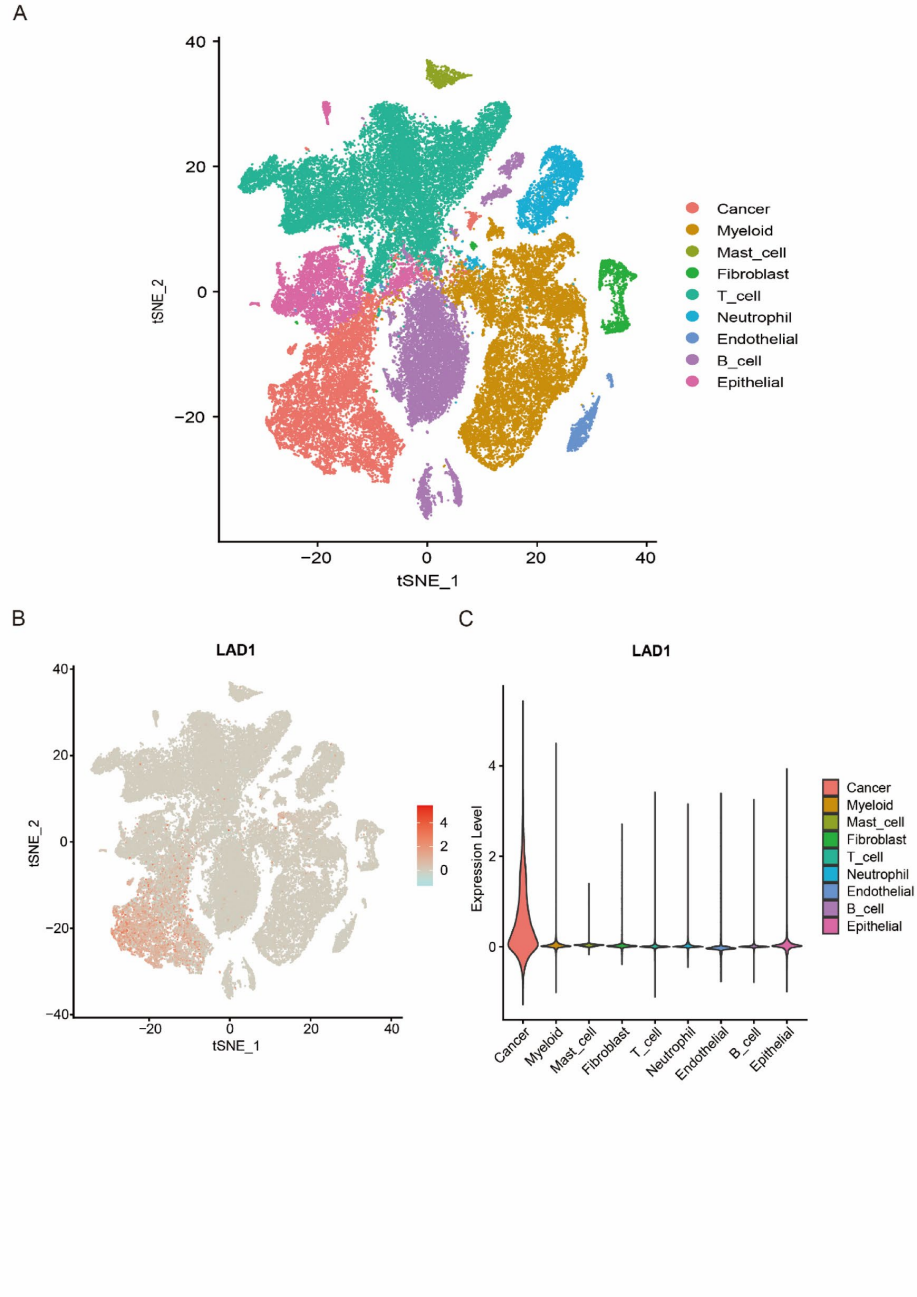
LAD1 was specifically expressed in the cancer cell subgroup of LUAD. (**A**) The t-SNE plot of LUAD scRNA-seq data. (**B**) The t-SNE plot of LAD1 expression in LUAD scRNA-seq data. (**C**) The relative levels of LAD1 in each cell subgroup of the LUAD.

Tumors are highly heterogeneous tissues comprising various cell types, such as cancer cells, endothelial cells, and immune cells. Traditional experimental methods face challenges in identifying specific cell type biomarkers within tumors. However, the application of scRNA-seq has greatly benefited the characterization of tumor composition and the discovery of cell type-specific markers. In this study, we analyzed scRNA-seq data from LUAD. The dataset included 58,685 cells obtained from tumors of 10 LUAD patients after quality control. By clustering the cells using a resolution of 0.5 and annotating cell subgroups with known markers, we identified nine cell subgroups, including cancer cells, myeloid cells, mast cells, fibroblasts, T cells, neutrophils, endothelial cells, B cells, and epithelial cells (Fig. 1A). Using the “FindMarkers” function, we discovered that LAD1 was specifically expressed in the cancer cell subgroup, with significantly higher expression compared to other cell types, including normal epithelial cells (Fig. 1B-C).

3.2 Altered DEGs, biological processes, and pathways due to expression variations of LAD1 in the cancer cell subgroup of lung adenocarcinoma.

**Fig. 2:**
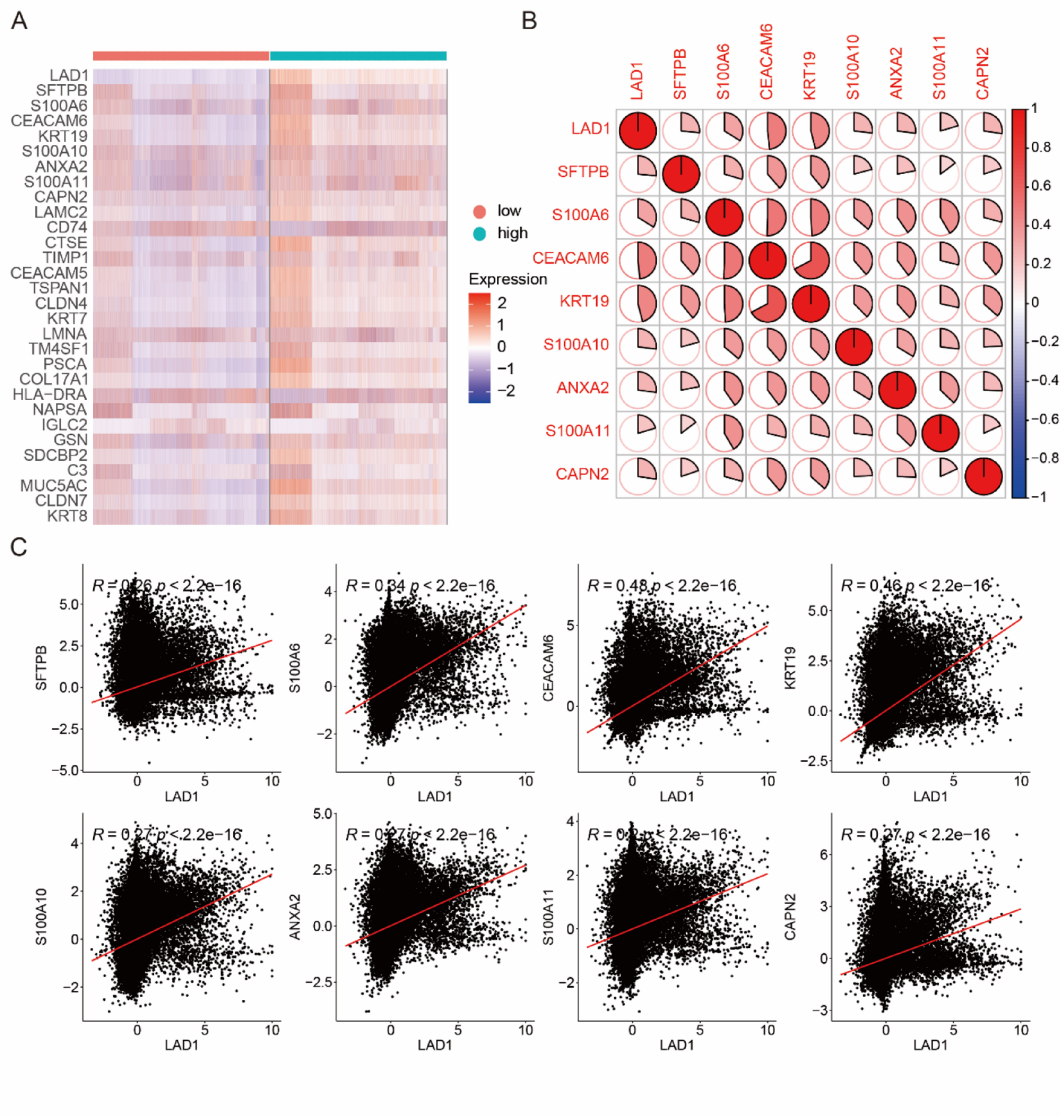
The DEGs between the high- and low-LAD1 expressing cancers in LUAD. (**A**) The heatmap of top altered DEGs in the LUAD scRNA-seq data. (**B**) The correlation between the top 9 altered DEGs. (**C**) The scatter plots between LAD1 and other top 8 altered DEGs.

**Fig. 3:**
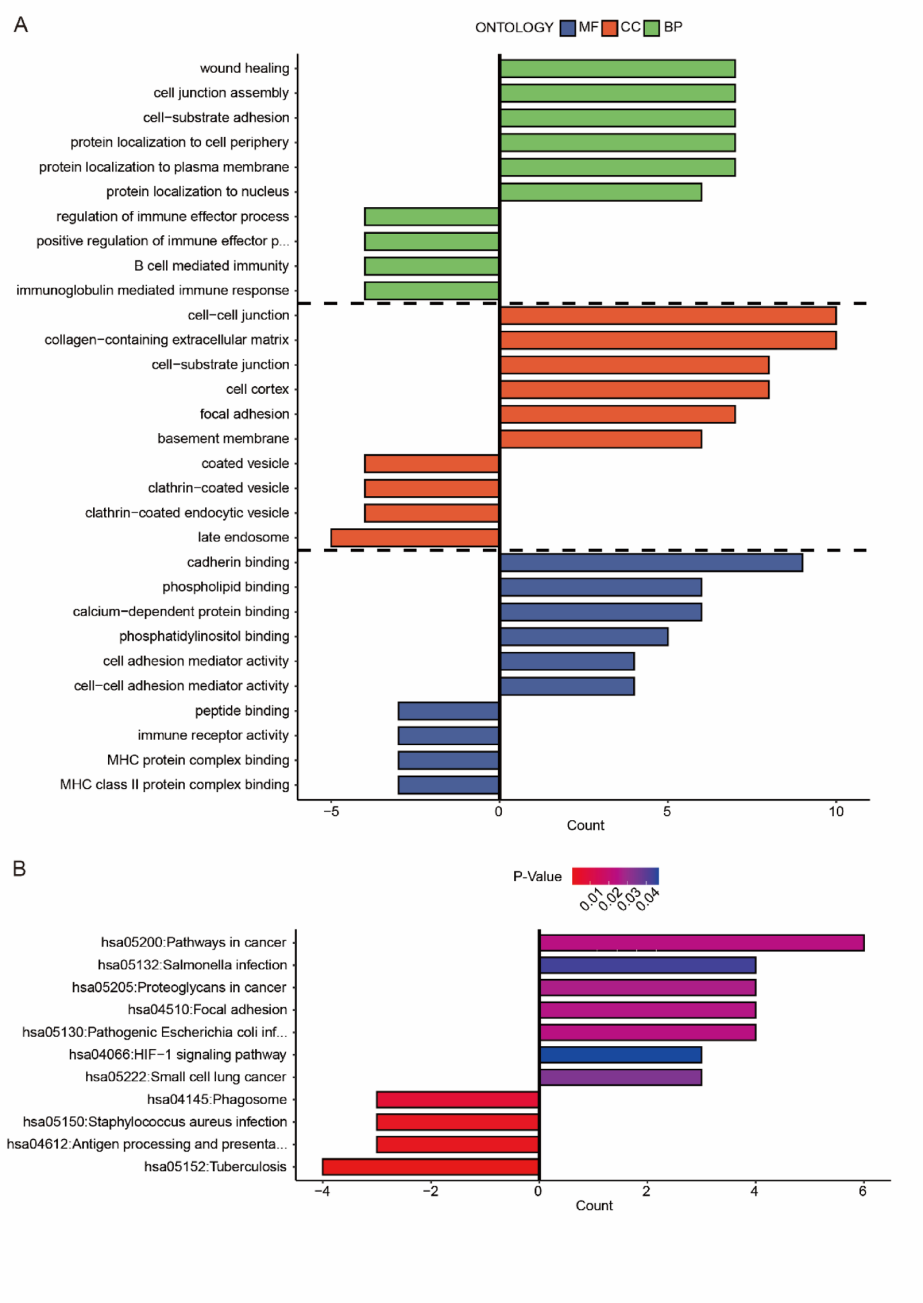
The (**A**) GO and (**B**) KEGG analyses of the DEGs between the high- and low-LAD1 expressing cancers in LUAD. Pathway analysis was performed using data from the Kyoto Encyclopedia of Genes and Genomes [*KEGG; Kanehisa Laboratories*, www.kegg.jp/kegg/kegg1.html*].*

Given that LAD1 was identified as a cancer cell-specific marker, we investigated whether its expression levels influenced gene expression, biological processes, and pathways within the cancer cell subgroup. Focusing on the cancer cell subgroups in the scRNA-seq data, we determined the differentially expressed genes (DEGs) between cancer cells with high and low LAD1 expression and visualized the top altered DEGs using a heatmap (Fig. 2A). We also examined the correlations between LAD1 and the top eight DEGs, including SFTPB, S100A6, CEACAM6, KRT19, S100A10, ANXA2, S100A11, and CAPN2. All correlations were statistically significant, with CEACAM6 and KRT19 showing the highest correlation coefficients with LAD1 (Fig. 2B-C). Notably, CEACAM6 is a metastasis biomarker, and its inhibition has been shown to improve the sensitivity of LUAD cancer cells to chemotherapy and anoikis^17–20^. KRT19 is associated with epithelial-to-mesenchymal transition (EMT) and poor prognosis in LUAD^21,22^. The strong correlations suggest that LAD1 may be involved in LUAD-related biological processes. Moreover, we conducted gene ontology (GO) analysis on the DEGs between cancer cells with high and low LAD1 expression, revealing the promotion of processes related to cell-cell junctions, cell-substrate junctions, and protein localization, while immune and vesicle-related processes were repressed (Fig. 3A). Pathway analysis indicated enhanced cancer-related and HIF-1 signaling pathways and suppressed phagosome and antigen processing and presentation pathways in cancer cells with high LAD1 expressions (Fig. 3B).

### Expressions and prognostic values of LAD1 in pan-cancer

**Fig. 4:**
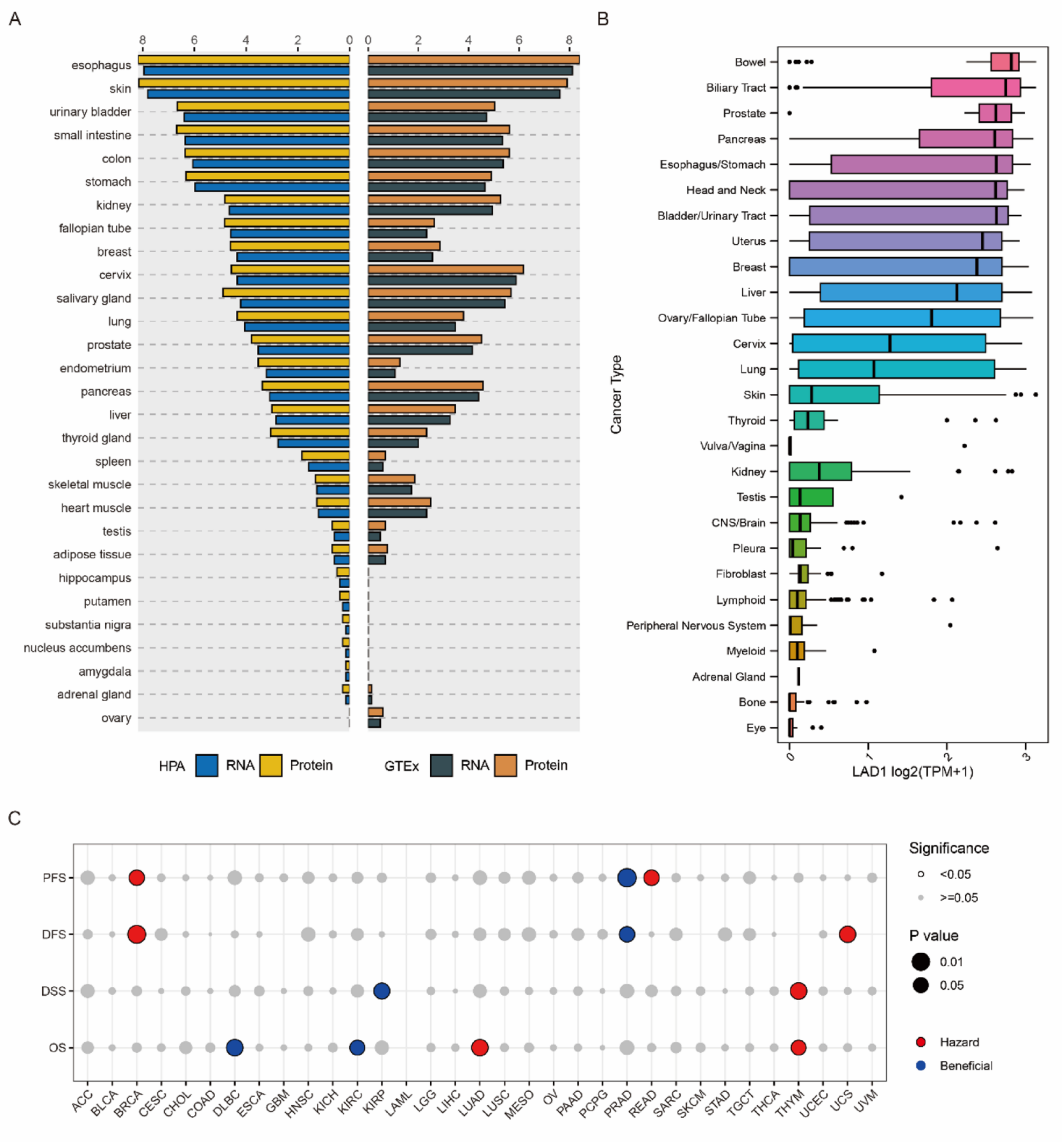
Pan-cancer analysis of LAD1 expression patterns and prognostic values. (**A**) The relative expressions of LAD1 in normal tissues. (**B**) The relative expressions of LAD1 in the cancer cell lines of different cancer types. (**C**) The prognostic values of LAD1 expression in all cancer types of the TCGA.

Considering the specific expression of LAD1 in LUAD cancer cells, we further explored its expression profiles across various tissues and cancers. LAD1 was found to be highly expressed in normal tissues such as the esophagus, skin, and urinary bladder (Fig. 4A). In terms of cancers, LAD1 showed high expression in bowel, biliary tract, prostate, and other cancer types (Fig. 4B). In lung tissues, LAD1 exhibited moderate expression in normal lung tissues, but its expression in lung cancer was relatively high compared to other types of cancer (Fig. 4A-B). We then investigated the prognostic value of LAD1 in pan-cancer. In terms of overall survival (OS), LAD1 was a significant hazard factor for LUAD and thymoma (THYM) but a beneficial factor for lymphoid neoplasm diffuse large B-cell lymphoma (DLBCL) and kidney renal clear cell carcinoma (KIRC). Regarding disease-specific survival (DSS), LAD1 was a significant hazard factor for THYM but beneficial for kidney renal papillary cell carcinoma (KIRP). As for disease-free survival (DFS), LAD1 was a significant hazard factor for uterine carcinosarcoma (UCS) and breast invasive carcinoma (BRCA) but beneficial for prostate adenocarcinoma (PRAD). In terms of progression-free survival (PFS), LAD1 was a significant hazard factor for BRCA and rectum adenocarcinoma (READ) but beneficial for PRAD (Fig. 4C).

### LAD1 upregulation in LUAD and its independent prognostic significance

**Fig. 5:**
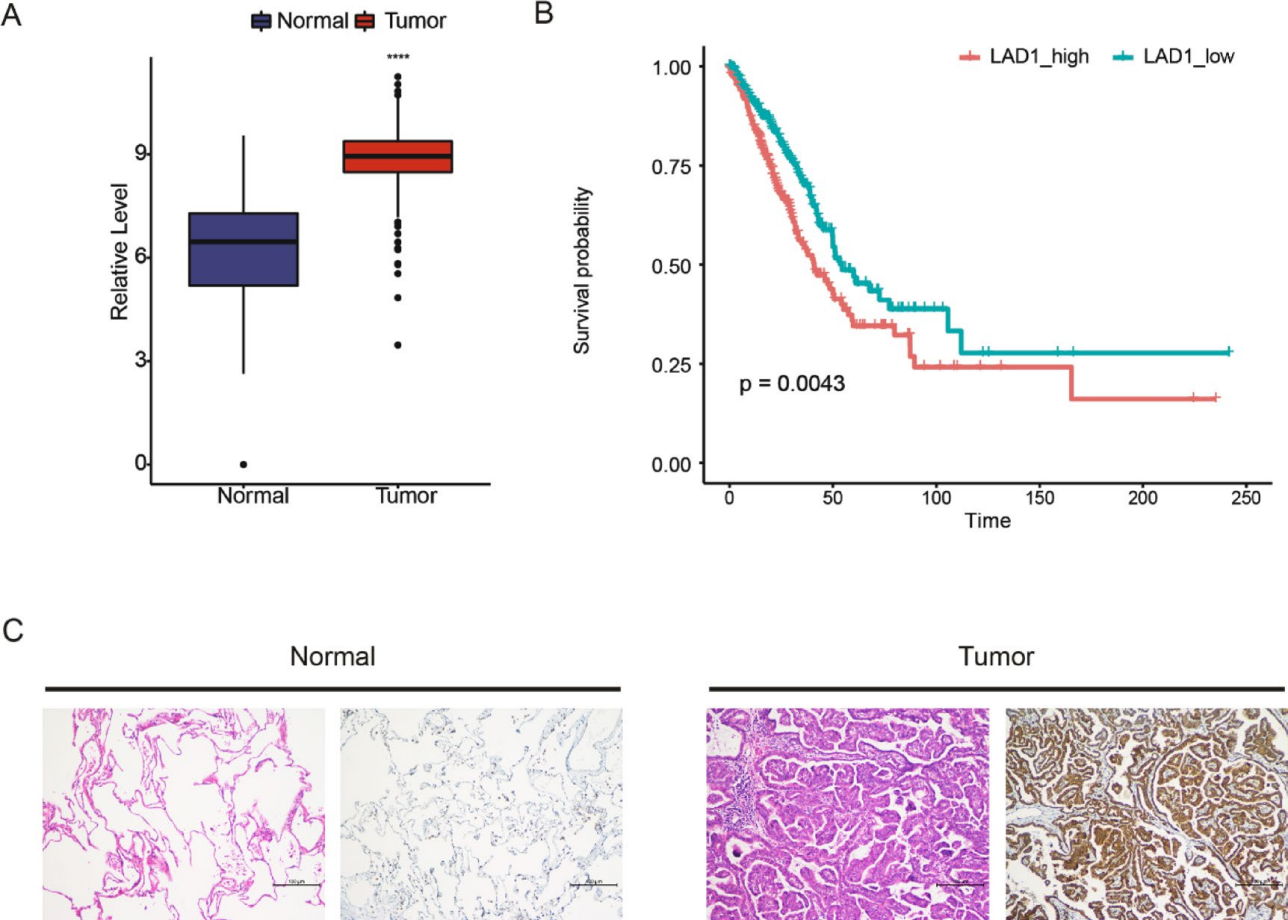
LAD1 was upregulated and associated with a poor prognosis of LUAD. (**A**) The boxplot plot of LAD1 expressions in normal lung and LUAD tissues. (**B**) The Kaplan-Meier survival curve of high- and low-LAD1 expressing patients. ****, *P* < 0.0001. (**C**) Immunohistochemical staining of LAD1 in lung adenocarcinoma and adjacent normal tissues.

**Fig. 6:**
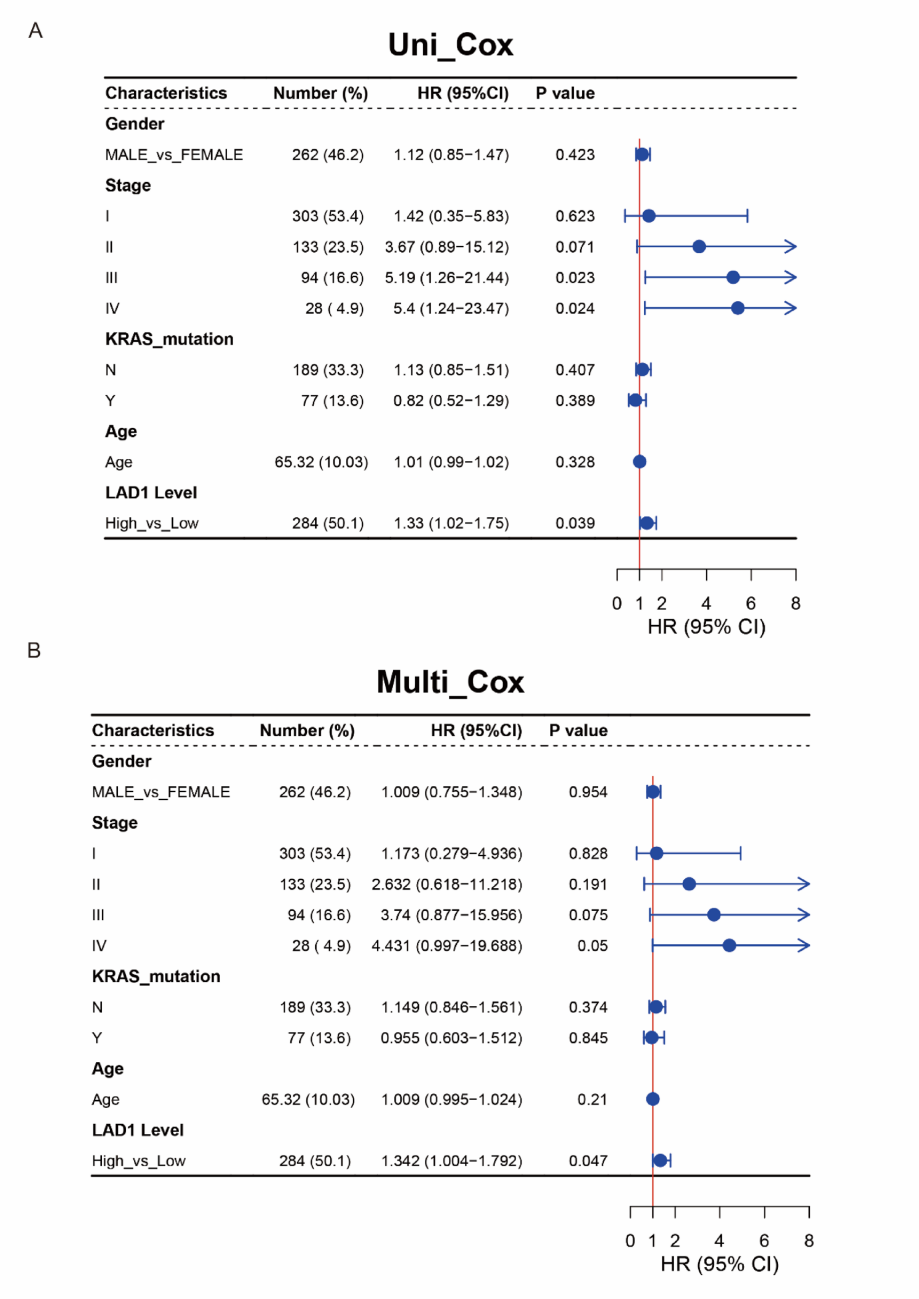
(**A**) Univariant and (**B**) multivariant Cox regression analyses showing that LAD1 was an independent prognostic factor for LUAD.

We investigated the expression changes of LAD1 in various cancer types compared to their corresponding normal tissues. LAD1 was significantly upregulated in LUAD and several other tumor types compared to normal tissues (Figure S1, Fig. 5A). Survival analysis confirmed that high LAD1 expression was associated with worse prognosis in LUAD (Fig. 5B). In order to investigate the relationship between LAD1 and the clinicalpathological characteristics of LUAD patients, the expression of LAD1 in LUAD tissues was detected by immunohistochemical staining. The results showed that the positive rate of LAD1 in LUAD cancer tissues was significantly higher than that in adjacent tissues (*P* < 0.001) (Fig. 5C; Table 2). Based on the analysis of clinical data (Table 3), it was found that the positivity rate of LAD1 in LUAD tissues was not significantly different with respect to patient age, gender, tumor location, grade, size, lymph node metastasis, and pleural invasion (*P* > 0.05). Furthermore, through univariate and multivariate Cox regression analyses, we assessed the independent prognostic significance of LAD1 in LUAD. Univariate analysis identified histological stage III and IV and LAD1 expression levels as significant hazard factors for LUAD (Fig. 6A). However, in the multivariate analysis, only LAD1 expression level remained as an independent prognostic factor (Fig. 6B).

**Table 2:** Expression of LAD1 protein in lung adenocarcinoma and adjacent tissues.

Characteristics	*N*	LAD1	
		Positive	Negative
Lung adenocarcinoma	36	25(69.4%)	11(30.6%)
Adjacent normal tissues	36	5(13.9%)	31(86.1%)
P-value		< 0.001^a^	

^a^:Chi-squared test.

**Table 3:** The relationship between the expression of LAD1 and clinical pathological features in lung adenocarcinoma.

Characteristics	LAD1_negative (*N* = 11)	LAD1_positive (*n* = 25)	*P*-value
Grade			
2	8 (72.7%)	23 (92.0%)	0.154^a^
3	3 (27.3%)	2 ( 8.0%)	
Location			
Left lung	6 (54.5%)	10 (40.0%)	0.483^a^
Right lung	5 (45.5%)	15 (60.0%)	
Pleural invasion			
0	9 (81.8%)	23 (92.0%)	0.570^a^
1	2 (18.2%)	2 ( 8.0%)	
pT			
1a	5 (45.5%)	12 (48.0%)	0.918^a^
1b	3 (27.3%)	8 (32.0%)	
1c	1 ( 9.1%)	2 ( 8.0%)	
2	2 (18.2%)	2 ( 8.0%)	
4	0 ( 0.0%)	1 ( 4.0%)	
pN			
NA	0 (0.0 %)	2 (8.0 %)	1.000^a^
0	10 (90.9%)	20 (80.0%)	
1	1 ( 9.1%)	3 (12.0%)	
Gender			
Female	8 (72.7%)	18 (72.0%)	1.000^a^
Male	3 (27.3%)	7 (28.0%)	
AGE			
<60	1 ( 9.1%)	10 (40.0%)	0.116^a^
≥60	10 (90.9%)	15 (60.0%)	

a: Fisher’s Exact Test, **pT**, pathologic tumor stage; **pN**, pathologic nodal stage. Staging was based on the 8th edition AJCC Cancer Staging Manual. *Note: * pN data were available for 34 patients; two patients did not undergo lymph node dissection.

### Knockdown of LAD1 expression in A549 cells using siRNA and detection of changes in LUAD cell migration and invasion ability

**Fig. 7:**
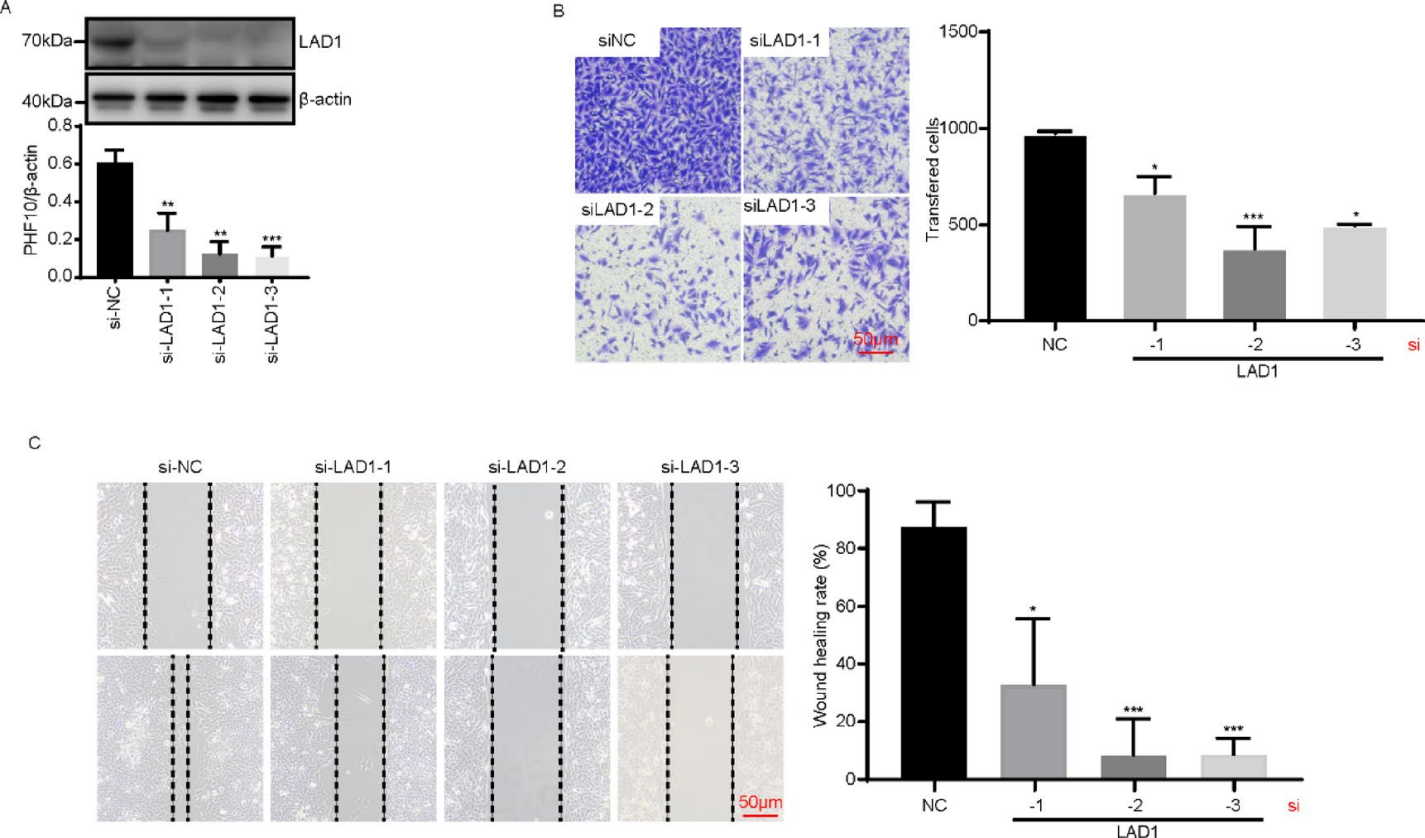
(**A**) Western blot detection of changes in LAD1 protein levels in A549 cells after transfection. (**B**) Transwell experiment detects the effect of knocking down LAD1 on the invasive ability of LUAD cells. (C) Scratch healing assay to detect migration ability of LUAD cells after knockdown of LAD1. Full-length blots are presented in Supplementary Figure S3. ∗ indicates *P* < 0.05;∗∗ indicates *P* < 0.01; ∗∗∗ indicates *P* < 0.001.

After transfecting the LUAD cell line A549 with siRNA, Western blot analysis revealed that, compared to the si-NC group, the LAD1 protein levels were significantly decreased in the si-LAD1#1, si-LAD1#2, and si-LAD1#3 groups of LUAD cells (*P* < 0.01 or *P* < 0.001) (Fig. 7A). These findings indicate the successful establishment of the LAD1 knockdown cell model.

The migration and invasion abilities of transfected LUAD cells were evaluated using scratch and Transwell assays. The scratch healing experiment (Fig. 7C) showed that compared with the si-NC group cells, the migration ability of LAD1-knockdown A549 cells was inhibited (*P* < 0.05 or *P* < 0.001). The Transwell invasion experiment results (Fig. 7B) showed that the invasion ability of A549 cells in the LAD1 knockdown group was lower than that in the si-NC group (*P* < 0.05 or *P* < 0.001). The above experimental results indicate that LAD1 knockdown inhibits the migration and invasion ability of LUAD cells.

### Differential gene profiles, biological processes, and signaling pathways associated with different LAD1 expressions

**Fig. 8:**
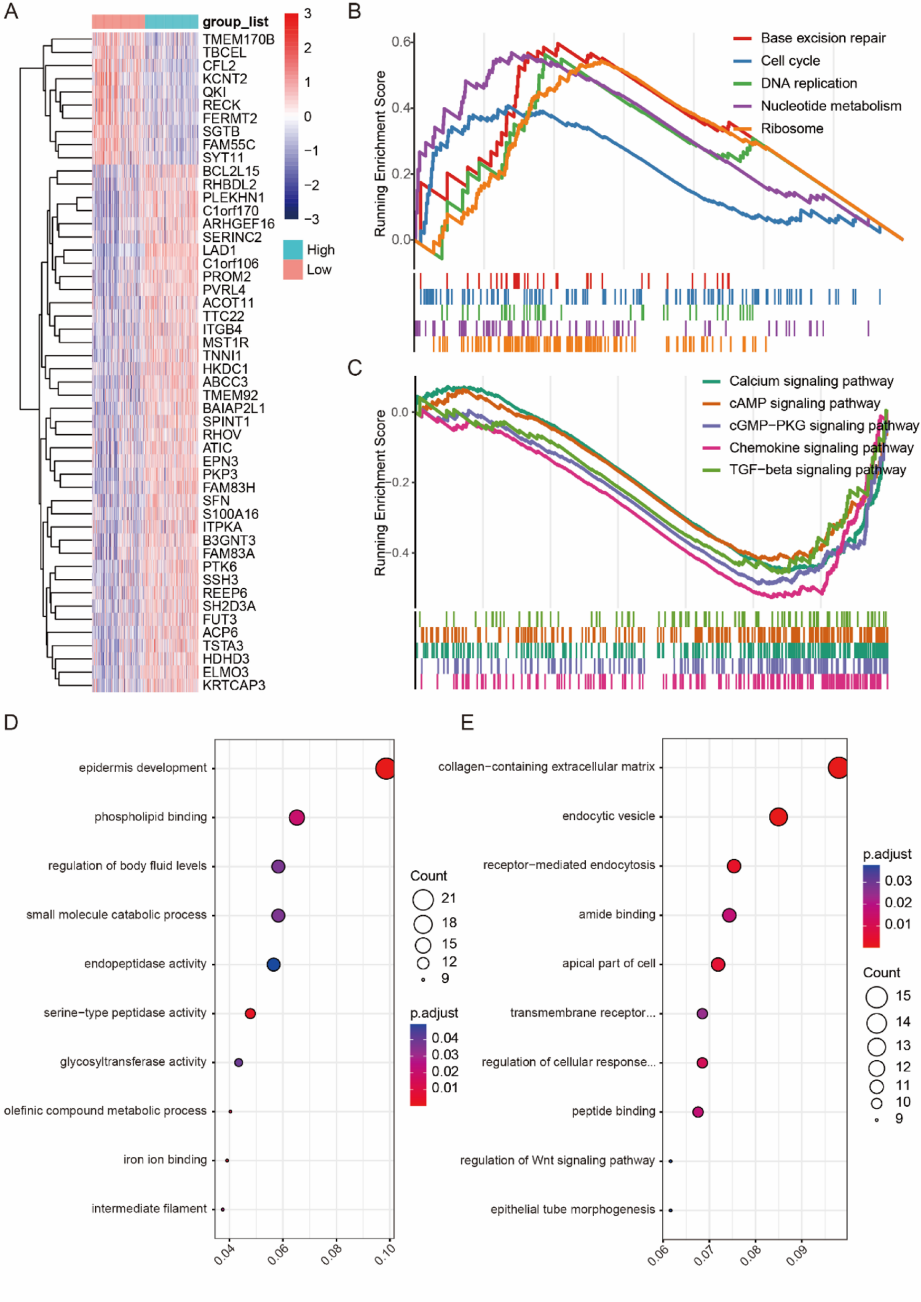
The gene profile differences between the LUAD patients with high- or low-LAD1 expressions. **(A)** The heatmap showing the relative levels of the top 50 level-changed DEGs. The (**B**) enhanced and (**C**) suppressed signaling pathways with high LAD1 expressions. The (**D**) enhanced and (**E**) suppressed GO terms with high LAD1 expressions.

To examine the gene expression changes resulting from LAD1 level variations, we analyzed both cancer cell-specific and whole tumor perspectives. We divided the LUAD cohort in TCGA into LAD1 high and low groups based on the median expression of LAD1 and identified 281 upregulated and 168 downregulated genes as differentially expressed between these groups. Heatmap visualization depicted the top 50 altered DEGs (Fig. 8A). Upregulated genes included PROM2, ITGB4, DKK1, and MMP10, while downregulated genes included BMP5, IL6, and TLR8. Furthermore, GSEA showed that high LAD1 expression was associated with enrichment of DNA replication and repair pathways, while suppressing immune-related pathways and signaling pathways (Fig. 8B-C). GO analysis further revealed functional enrichment in cell adhesion and metabolic processes in LAD1-high samples (Fig. 8D-E).

### Impact of LAD1 levels on immune cell infiltrations and their association with LUAD prognosis

**Fig. 9:**
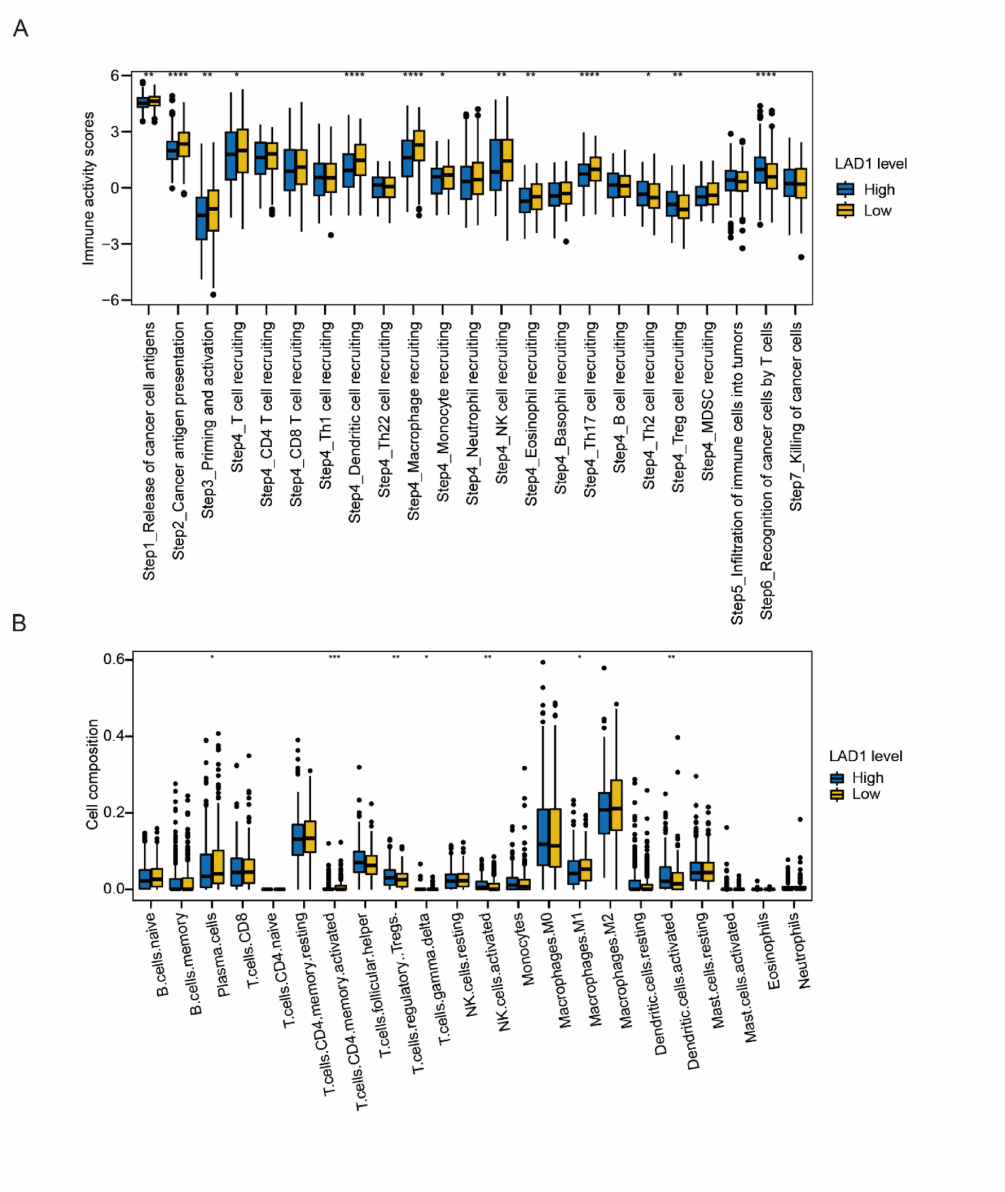
The association of LAD1 and immune cell infiltrations in LUAD. (**A**) The content changes of anti-cancer immune responses with altered LAD1 expressions. (**B**) The changes of immune cell infiltrations that were affected by altered LAD1 expressions. *, *P* < 0.05; **, *P* < 0.01; ***, *P* < 0.001; ****, *P* < 0.0001.

Immune cell responses and infiltrations play a crucial role in the growth, survival, and metastasis of cancer cells. We investigated the changes in immune cell responses by performing single-sample gene set enrichment analysis (ssGSEA) on the TCGA-LUAD cohort. Comparing immune cell response steps between the LAD1 high and low groups, we found significant upregulation in the release of cancer cell antigens, cancer antigen presentation, and priming and activation, while recognition of cancer cells by T cells was significantly downregulated by LAD1. Regarding the trafficking of immune cells to tumors, the effect of LAD1 varied among immune cell types, promoting the recruitment of T cells, dendritic cells, macrophages, monocytes, NK cells, eosinophils, and Th17 cells, while suppressing the recruitment of Th2 and Treg cells. However, immune cell infiltration into tumors and killing of cancer cells were not significantly changed (Fig. 9A).

To further investigate immune cell infiltrations, we performed CIBERSORT analysis and observed increased infiltrations of plasma cells, activated memory CD4 T cells, and M1 macrophages in the LAD1-high group, whereas Tregs, γδT cells, activated NK cells, and activated dendritic cells were significantly decreased (Fig. 9B). Notably, changes in immune cell infiltrations due to LAD1 levels did not lead to significant alterations in LUAD overall survival (Figure S2). These findings suggest that while LAD1 affects certain immune cell infiltrations, they may not be responsible for the observed prognosis alterations in LUAD.

### LAD1 as a prognostic predictor for LUAD

**Fig. 10:**
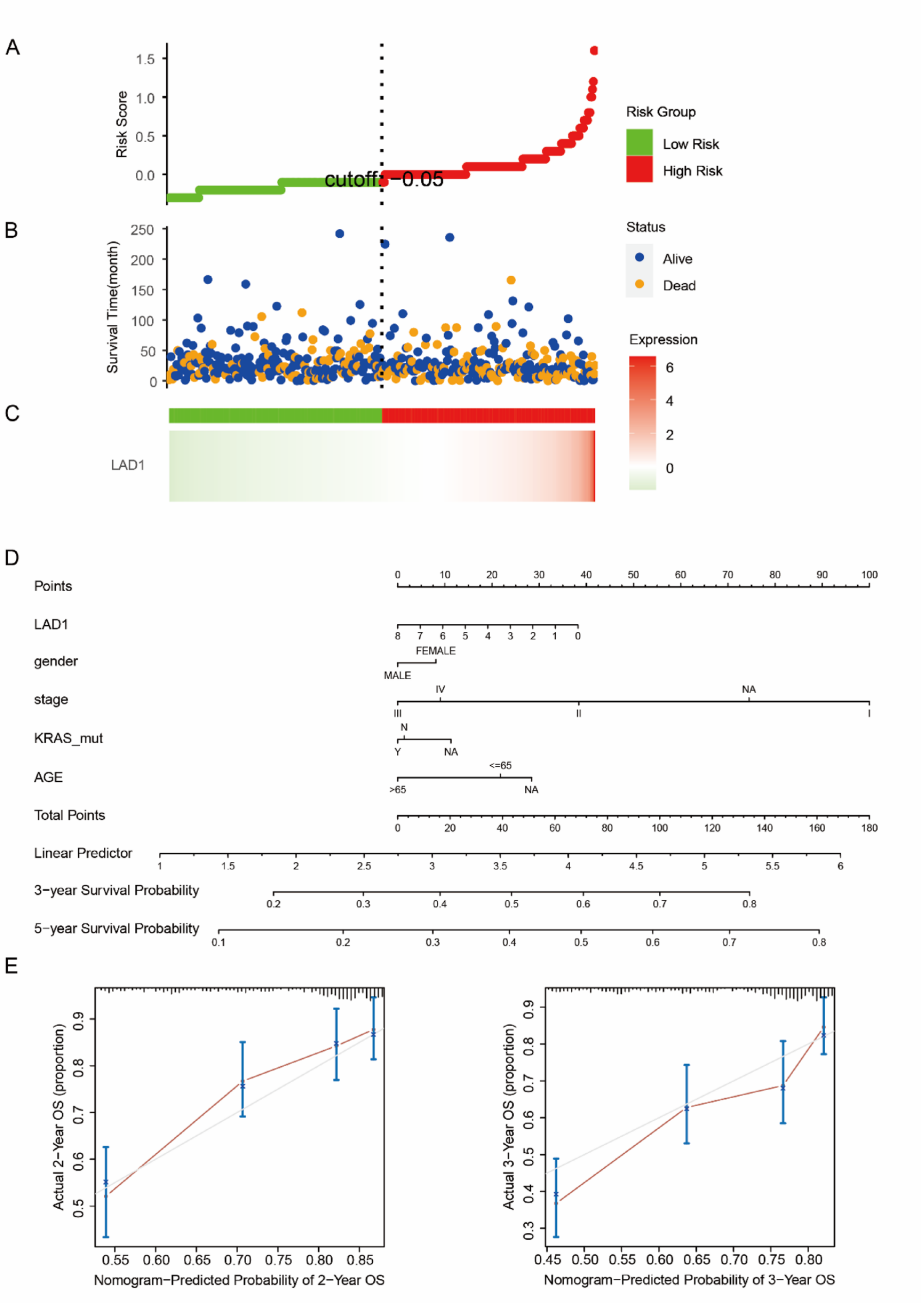
LAD1 as a predictive factor for LUAD prognosis. (**A**–**C**) The association of LAD1 expressions with risk and survival in LUAD. (**D**) A nomogram model was constructed with LAD1 expressions and other clinical parameters. (**E**) The validation of the nomogram model using the actual 2- and 3-year OS data.

**Fig. 11:**
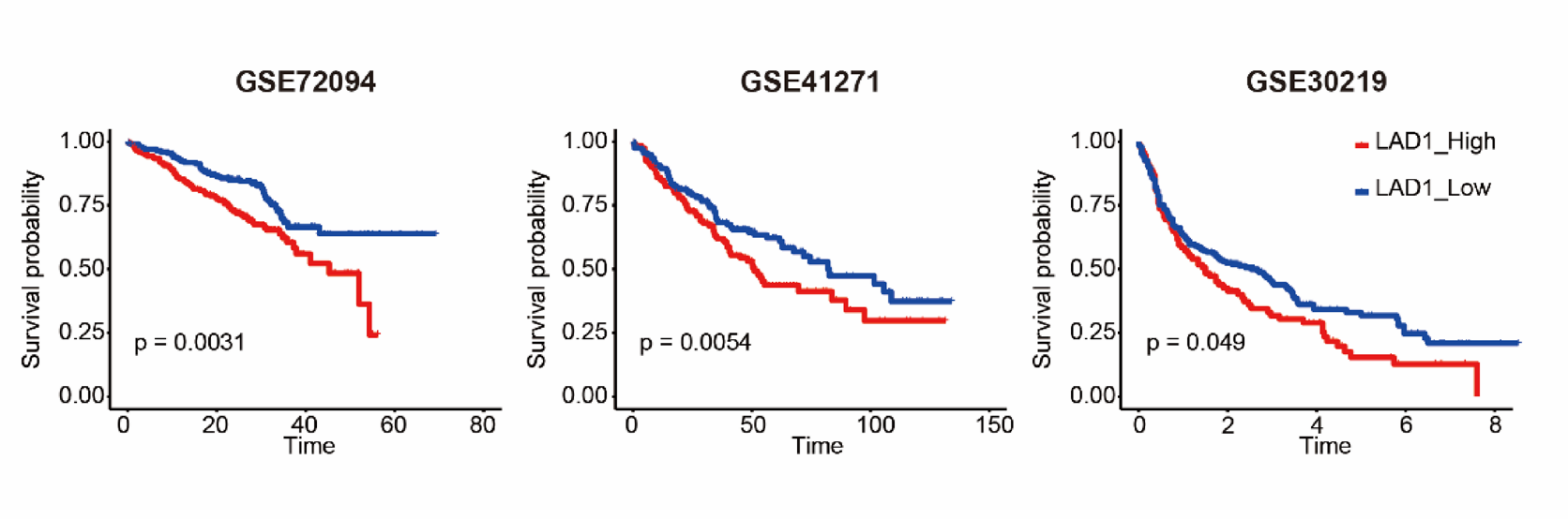
The prognostic value of LAD1 in LUAD was verified with validation cohorts. Patients were divided based on the median values of LAD1 expressions in (**A**) GSE72094, (**B**) GSE41271, and (**C**) GSE30219, respectively.

Given the aberrant upregulation of LAD1 in LUAD and its association with poor prognosis, we explored its potential as a prognostic predictor. Using univariate Cox analysis, we calculated risk scores based on LAD1 expressions, where high LAD1 expression indicated high risk (Fig. 10A and C). The high-risk group showed more deaths and shorter survival intervals compared to the low-risk group (Fig. 10B). These results indicate that LAD1 can be used to predict general risks in LUAD. We further performed a nomogram analysis incorporating LAD1 levels, gender, histological stages, KRAS mutations, and ages in LUAD to construct a prognostic model. High LAD1 expression, advanced histological stages, KRAS mutations, and older age contributed to a relatively worse prognosis, consistent with previous reports and our study findings (Fig. 10D). Furthermore, calibration of the nomogram model using actual 2-year and 3-year overall survival (OS) data demonstrated its accurate prediction capabilities (Fig. 10E). To validate the prognostic value of LAD1 beyond the TCGA-LUAD cohort, Kaplan-Meier survival curves were plotted for three independent validation cohorts (GSE72094, GSE41271, and GSE30219), all indicating that high LAD1 expressions were associated with significantly shorter OS (Fig. 11).

## Discussion

Lung adenocarcinoma (LUAD) is a major subtype of non-small cell lung cancer (NSCLC) and is associated with poor prognosis. Despite the availability of multiple treatment approaches, there is a need to identify novel factors that are involved in LUAD tumorigenesis and prognosis. Tumors are highly heterogeneous tissues composed of various cell types, and the use of single-cell RNA sequencing (scRNA-seq) technology enables the characterization of gene expression profiles within tumors at a single-cell resolution. In this study, we applied scRNA-seq to investigate the expression pattern of LAD1 in LUAD. Our findings revealed that LAD1 was highly expressed in cancer cells, highlighting its potential significance in LUAD. In order to further verify the high expression of LAD1 in LUAD tissues, immunohistochemical staining was performed on cancer tissues and corresponding adjacent tissues collected from 36 LUAD patients. The results revealed a significant difference in LAD1 expression between the cancerous and adjacent tissues, indicating that LAD1 may have potential diagnostic and prognostic relevance in LUAD. In order to investigate the effect of LAD1 on the function of LUAD cells, this study used siRNA to knock down LAD1 and found that LAD1 knockdown could inhibit the migration and invasion of LUAD cells, suggesting that LAD1 may be involved in LUAD progression through effects on tumor cell migration and invasion. Overall, LAD1 may be involved in the occurrence and development of LUAD, but its mechanism of action still needs further exploration. The high expression of LAD1 in cancer cells led us to explore its potential associations with genes previously implicated in LUAD tumorigenesis. Through correlation analysis, we observed that LAD1 expression levels were statistically associated with several cancer-related genes, including SFTPB, S100A6, CEACAM6, KRT19, S100A10, ANXA2, S100A11, and CAPN2. Although these associations do not establish causality, the consistent co-expression patterns may suggest that LAD1 is part of a broader regulatory network relevant to LUAD biology, warranting further experimental investigation. Our findings suggest that LAD1 may be associated with pathways involving these genes during LUAD tumorigenesis.

LAD1 encodes a basement membrane filament protein, which functions to maintain the association between epithelial cells and the mesenchyme and thus regulates the epithelial-to-mesenchymal transition (EMT) and cell migration^8,9^. LAD1 was found to be a tumorigenesis factor in multiple cancers, such as colorectal cancer, prostate cancer, breast cancer, etc^10–13^. As to LUAD, the detailed expression distribution and its downstream pathways were still unclear. Therefore, we investigated the impact of LAD1 expression on biological processes and pathways at both the single-cell and whole-tumor levels. Interestingly, we observed distinct differences in the affected processes and pathways between these two levels, which could be attributed to the complex interactions between various cell types within tumors. Nevertheless, both analyses revealed that alterations in LAD1 expression may influence processes relevant to tumorigenesis.

Furthermore, we investigated the LAD1-correlated genes in the LUAD cancer cell subgroup, among which SFTPB, S100A6, CEACAM6, KRT19, S100A10, ANXA2, S100A11, and CAPN2 displayed top correlations with LAD1. These genes were previously demonstrated as important cancer regulatory genes. SFTPB was also an independent biomarker for lung cancer diagnosis^23^. S100A6 was bifacial in lung cancer: it inhibited lung cancer cell growth, but its upregulation predicted a poor prognosis of NSCLC^24,25^. CEACAM6 led to enhanced Src-FAK pathway activation and chemotherapy resistance, making it a potential therapeutic target of LUAD^17,18,20^. KRT19 was upregulated in LUAD and showed a relationship with EMT-related gene sets, as well as with poor prognosis^22^. S100A10 promoted the proliferation and metastasis through the AKT/mTOR pathway in LUAD^26^. ANXA2 was a circRNA that facilitated proliferation and metastasis via regulating PDPK1 in lung cancer^27^. CAPN2 promoted cancer progression and chemotherapy resistance through the EGFR/AKT pathway^28^. In summary, LAD1 might be linked to these genes and could participate in LUAD-associated biological pathways.

The prognostic value of LAD1 was explored through pan-cancer analyses. Our results indicated that LAD1 was a significant hazard factor for overall survival (OS) in LUAD and thymoma (THYM), while showing beneficial effects for lymphoid neoplasm diffuse large B-cell lymphoma (DLBC) and kidney renal clear cell carcinoma (KIRC). Cox regression and Kaplan–Meier analyses confirmed LAD1 as an independent prognostic hazard factor for LUAD, and these findings were validated using independent cohorts. Moreover, we developed a nomogram model incorporating LAD1 expression and other clinical parameters, which showed good predictive performance and demonstrated reasonable agreement with clinical outcomes.

This study has several limitations. The immunohistochemistry cohort included a relatively small number of cases, and the in vitro functional assays were performed in only one LUAD cell line, which may not fully represent the biological heterogeneity of the disease. In addition, the mechanistic roles of LAD1 were not investigated in vivo, and the correlations identified through bioinformatic analyses cannot establish causality. Therefore, studies incorporating larger patient cohorts and more comprehensive in vitro and in vivo experiments are needed to further clarify the biological significance of LAD1 in LUAD.

## Conclusions

In summary, LAD1 expression is increased in LUAD tissues, and knocking down LAD1 can significantly inhibit the migration and invasion of LUAD tumor cells. LAD1 may be involved in LUAD progression through its effects on tumor cell migration and invasion. Additionally, the prognostic value of LAD1, as demonstrated by its association with overall survival, highlights its significance in predicting patient outcomes. The nomogram model incorporating LAD1 expression and clinical parameters provides a useful tool for LUAD prognosis prediction. There is relatively little research on LAD1, and our study only conducted a preliminary exploration of its expression, migration, and invasion in LUAD, based on a limited number of cases. In-depth research on LAD1 and its regulatory mechanisms may provide new insights into its biological role in LUAD.

## Supplementary Information

Below is the link to the electronic supplementary material.

Supplementary Material 1

Supplementary Material 2

Supplementary Material 3

Supplementary Material 4

## Data Availability

Public data analyzed in this study can be retrieved from the following databases:-**GEO**: GSE136246, GSE72094, GSE41271, GSE30219 (https://www.ncbi.nlm.nih.gov/geo/), **GTEx**: (https://gtexportal.org/), **HPA**: (https://www.proteinatlas.org/), **CCLE**: (https://portals.broadinstitute.org/ccle/), **TCGA**: (https://portal.gdc.cancer.gov/).
