# Supplementary figures and images for: Exploring the expression and prognostic roles of LAD1 in lung adenocarcinoma
Source: Sci Rep. 2025 Dec 21;15:45124. doi: 10.1038/s41598-025-33277-z (PMC12749629; doi:10.1038/s41598-025-33277-z)

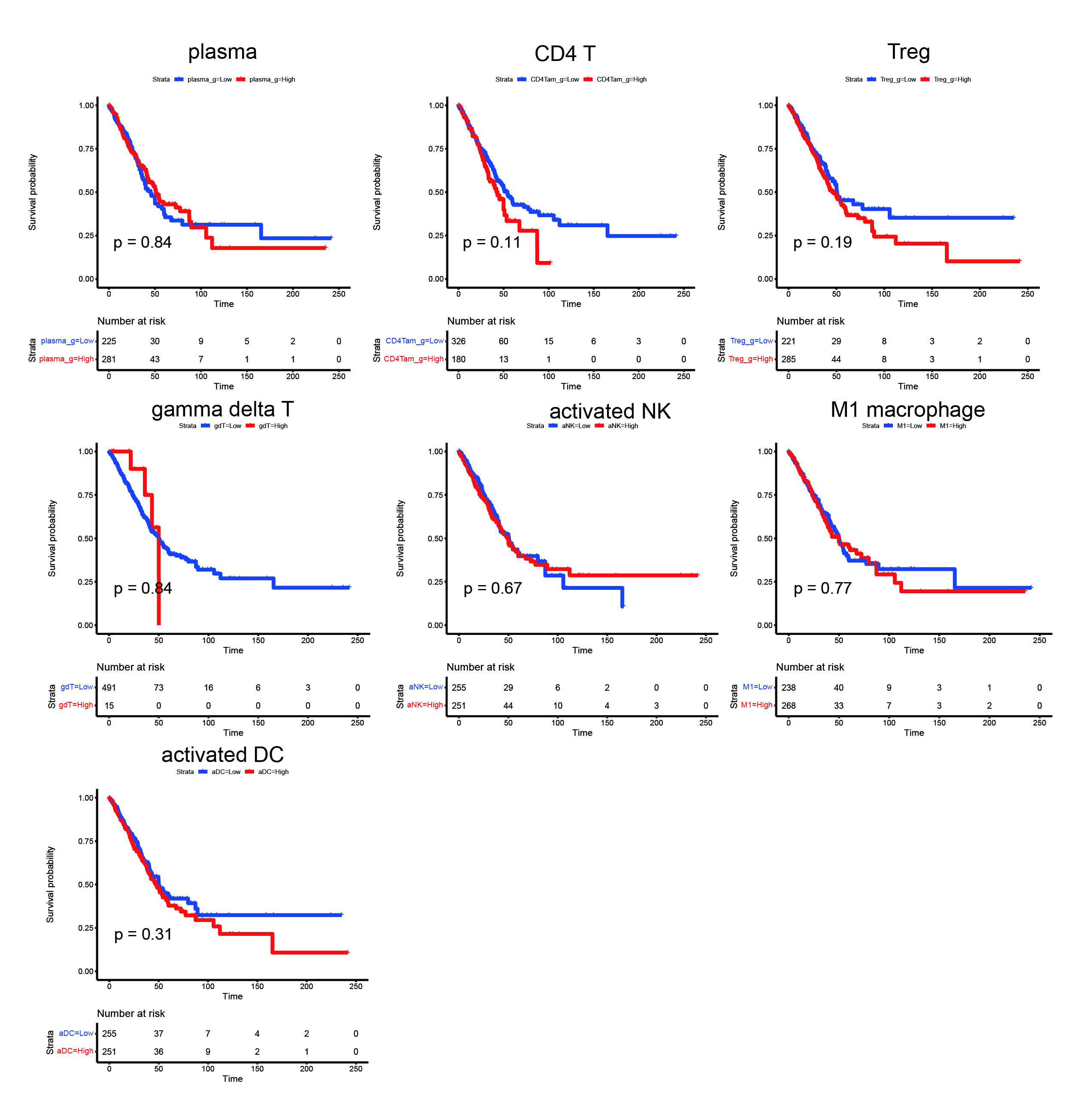

Supplement: Supplementary file 1 — Supplementary Material 1 [file 41598_2025_33277_MOESM1_ESM.tif]

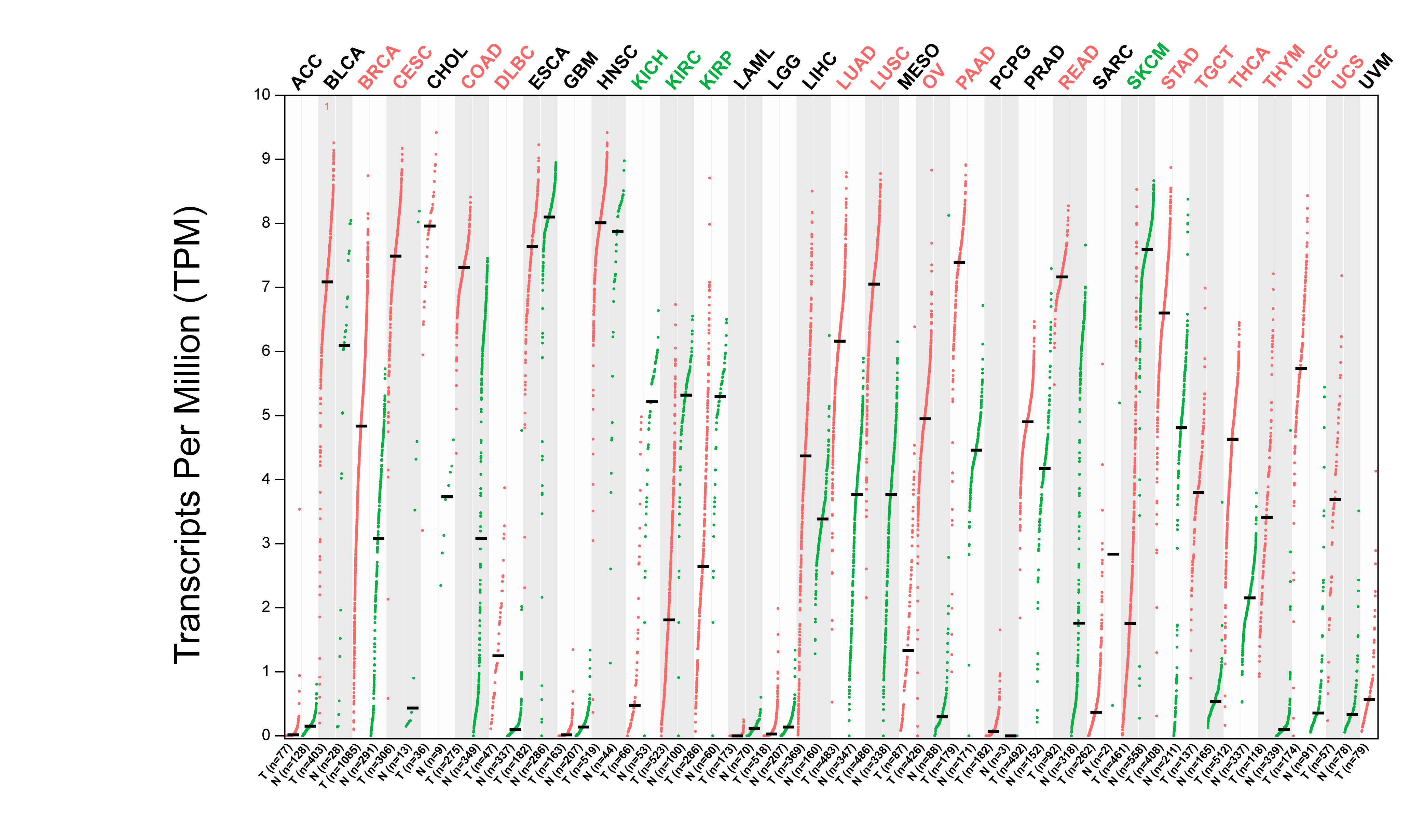

Supplement: Supplementary file 2 — Supplementary Material 2 [file 41598_2025_33277_MOESM2_ESM.tif]
