# Supplementary material for: Exploring the expression and prognostic roles of LAD1 in lung adenocarcinoma
Source: Sci Rep. 2025 Dec 21;15:45124. doi: 10.1038/s41598-025-33277-z (PMC12749629; doi:10.1038/s41598-025-33277-z)
Supplement: Supplementary file 3 — Supplementary Material 3 [file 41598_2025_33277_MOESM3_ESM.docx]

**Figure S1.** The relative expressions of LAD1 in all cancer types of TCGA and their corresponding normal tissues.

**Figure S2.** The Kaplan-Meier survival curves showing the prognostic values of immune infiltrations that were significantly adjusted by LAD1 dysregulations.

**Figure S3.** The uncropped, original full-length Western blot images corresponding to Figure 7A.
